# Effects of Dietary Protein Levels on Digestion, Metabolism, Serum Biochemical Indexes, and Rumen Microflora of Lanzhou Fat-Tailed Sheep

**DOI:** 10.3390/ani15010025

**Published:** 2024-12-25

**Authors:** Na Jiao, Wangmei Feng, Chi Ma, Honghe Li, Junsong Zhang, Juanshan Zheng, Penghui Guo

**Affiliations:** School of Life Science and Engineering, Northwest Minzu University, Lanzhou 730030, China; jiaona0812@163.com (N.J.); y230830438@stu.xbmu.edu.cn (W.F.); 19838206117@163.com (C.M.); 15077630313@163.com (H.L.); 15186867811@163.com (J.Z.)

**Keywords:** Lanzhou fat-tailed sheep, dietary protein levels, digestion and metabolism, serum biochemical indexes, rumen microflora

## Abstract

This study aimed to investigate the effect of varying levels of dietary protein on digestion, metabolism, serum biochemical indexes, and rumen microflora in Lanzhou fat-tailed sheep. A total of twenty 8-month-old healthy rams with an initial average body weight (BW 25.16 ± 1.09 kg) were selected and randomly divided into four dietary treatments with different protein levels P: 9.47%, MP: 10.53%, HP: 11.56%, and EHP: 12.61%. The rams underwent a 23 day adaptation period, and the experiment was conducted for 7 d. The results showed that with increased dietary protein levels, the apparent digestibility of dry matter (DM), crude protein (CP), acid detergent fiber (ADF), and urine energy significantly increased (*p* < 0.05), increasing by 8.65%, 7.31%, 6.22%, and 0.1%, respectively. In contrast, the digestibility of neutral detergent fiber (NDF) first increased and then plateaued with the increase in protein levels, and the digestibility of NDF in the HP group was the highest (*p* < 0.05). The Shannon, Simpson, Chao1, and Ace indices of rumen microorganisms in the four groups did not change significantly (*p* > 0.05). Additionally, at the phylum level, the relative abundance of Bacteroidota, Firmicutes, and Verrucomicrobiota collectively accounted for over 91% of the total phylum composition. At the genus level, there was an increase in the relative abundance of uncultured_rumen_bacterium and Rikenellaceae_RC9_gut_group in the LP group, whereas the relative abundance of Prevotella decreased. In comparison to the other three groups, the HP group exhibited an increase in the relative abundance of Firmicutes. In summary, dietary protein level had no significant effects on the rumen microflora structure and blood biochemical indexes. However, diets with a high protein level can improve the apparent digestibility of nutrients and energy use efficiency of Lanzhou fat-tailed sheep.

## Simple Summary

Dietary protein levels serve as the fundamental factor influencing sheep growth, development, and production performance. It significantly affects sheep digestion, metabolism, and rumen microbial diversity. Lanzhou fat-tailed sheep, recognized as a national endangered species and a valuable local sheep germplasm resource in China, represent a significant component of the country’s sheep germplasm resource strategy. However, there are currently no relevant reports addressing the protein requirements of Lanzhou fat-tailed sheep, nor is there a systematic feeding standard established for this breed. Therefore, the objective of this research was to investigate the effects of different protein levels in the diet on digestion, metabolism, serum biochemical indexes, and rumen microbial diversity in Lanzhou fat-tailed sheep. This research provides a theoretical basis and data support for the conservation and innovative utilization of Lanzhou fat-tailed sheep germplasm resources.

## 1. Introduction

Lanzhou fat-tailed sheep have the advantages of crude feed tolerance, higher disease resistance, and higher resilience [1]. Dietary protein is a critical nutrient that directly influences the health and production of livestock [2]. The levels of digestibility of ADF and NDF rise in tandem with increases in dietary protein [3]. Studies have indicated that augmenting the protein level in the lactation diet of Hu sheep can result in amplified milk production in ewes, along with a heightened carcass weight and offspring slaughter rate [4,5]. Goats’ growth performance can be affected by varying protein levels; high protein levels can greatly increase the average daily weight gain of goats [6]. Prado et al. [7] recommended using diets with a high protein level (16.8%) and a high lysine/methionine ratio (3.4) in animals slaughtered at a young age in order to obtain carcasses with a high muscle content without negatively affecting productive traits or intramuscular fat composition. Additionally, Wang et al. [8] found that a higher dietary CP level promoted growth performance for finishing lambs, whereas a lower dietary CP level was beneficial for meat quality, especially when evaluating color characteristics in the final product. In conclusion, the animal production and economic benefits of animal husbandry can be improved by a comprehensive understanding of the protein requirements of animals.

Insufficient protein supply will inhibit the growth of lean meat. On the contrary, if protein is supplied too much, excess protein will be deaminated and excess nitrogen will be excreted, which will not only waste protein resources but also adversely affect animal health [9]. Therefore, it is of great significance to formulate appropriate protein demand standards for improving livestock production performance, saving feed resources, and protecting the environment. At present, the protein requirement standards of Dorper × Hu crossbred sheep [10] and Santa Ines crossbred sheep [11] have been formulated. However, the protein requirements of different breeds of mutton sheep are different, and there is a lack of research on the protein requirements of Lanzhou fat-tailed sheep. Hence, the objective of this study was to evaluate the effects of feeding different levels of dietary protein to Lanzhou fat-tailed sheep on their digestion, metabolism, serum biochemical indexes, and rumen microflora in order to screen out the appropriate protein level for growth of Lanzhou fat-tailed sheep. In this study, a protein level of 11.56% could improve the apparent digestibility of nutrients and energy utilization efficiency. Therefore, under the conditions of this experiment, the appropriate protein level of the Lanzhou fat-tailed rams was 11.56%.

## 2. Materials and Methods

### 2.1. Ethics Committee Approval

This study was approved by the Animal Welfare Committee of Northwest Minzu University, Lanzhou, China (XBMU-SM-2020010). These procedures adhered to the principles and regulations for ethical protection in human and animal biological science and technology in China.

### 2.2. Experimental Design and Animals

This study was conducted in a National Lanzhou fat-tailed sheep breeding farm, located in Yongjing County, Linxia Hui Autonomous Prefecture, Gansu Province, China. The experiment was evaluated the effects of sheep fed four types of fodder. Twenty 8-month-old rams [BW, 25.16 ± 1.09 kg] with similar body conditions were selected. Before the commencement of the experiment, all animals were dewormed. Twenty rams were randomly divided into four groups, with five replicates in each group and one sheep in each replicate. The experiment lasted for 30 days, including 23 days of dietary adaptation and 7 days of digestibility measurement. Each ram was placed in a metabolic cage. To lessen the possibility that illnesses would affect the experiment’s results, a thorough cleaning of their shed and metabolic cages was performed before the experiment.

### 2.3. Diet and Feeding Management

The diet formula was based on China’s feeding standard (NY/T816-2021) [12]. All four diets contained roughage (wheat straw, alfalfa hay) and concentrate (corn, soybean meal, wheat bran), and the contents of protein were 9.47% (LP group), 10.53% (MP group), 11.56% (HP group), and 12.61% (EHP group), respectively. The dietary composition of the experimental groups is detailed in Table 1. Sheep feeding was carried out from 7 am to 6 pm each day. All rams were under restricted feeding conditions. Clean water was freely available at all times.

### 2.4. Measurements and Sample Collection

All rams were weighed every seven days. These measurements were recorded as the initial body weight (IBW), the final weight (FBW) From the IBW, FBW, and the weight recorded every seven days, the live weight gain (LWG) was subsequently calculated. Each sheep’s daily feed and residual feed were recorded for seven days, and feed refused was sampled daily for each ram. Then, the samples were dried at 105 °C for 4 h in a forced-air oven followed by equilibration in a desiccator and weighed for DMI analysis.

After the start of the digestibility measurement, 0.5 kg of concentrate and coarse material from the different experimental groups were collected, packed into Ziplock bags, marked, and stored in the refrigerator at −20 °C for later use. After mixing the dietary samples collected in each phase of the test, samples of appropriate proportions were taken and dried to constant weight in an oven at 105 °C. After the samples were cooled, they were crushed by a pulverizer and passed through a 40-mesh sieve for later use.

The rams were placed in a metabolic cage, under which a stool collection dish was placed to collect feces. Fecal samples were collected by the total fecal collection method before daily morning feeding for 7 consecutive days during the digestibility test. A 10% H_2_SO_4_ solution was mixed with 20% of the total daily fecal output, and a 10% HCl solution was mixed with 10% of the total daily urine output, which was then stored at −20 °C for the later determination of apparent nutrient digestibility.

After overnight fasting on the 30th day, 10 mL of blood was collected via the jugular vein. Blood samples obtained from each ram were placed into plain tubes and then centrifuged at 3000× *g* for 15 min at 4 °C to collect serum, which was stored at −20 °C for further analysis.

Rumen fluid was collected on the last day of the experiment using an oral stomach tube. Briefly, rumen fluid (70 mL/animal, liquid part) was collected using an oral stomach tube from each animal before morning feeding and snap-frozen in liquid nitrogen and then stored at −80 °C until use. Each time before taking the new sample, the tube was thoroughly cleaned with fresh warm water, and about 10–15 mL of the sample from each sheep was always discarded to prevent saliva contamination.

### 2.5. Digestion and Metabolism Analysis

The feed and fecal samples were dried to a constant weight at 105 °C using an oven. Crude protein (CP) was determined by a Kjeldahl apparatus (K9840, Shandong Haineng, China). Crude fat (EE) was determined by a fat analyzer (SOX406, Shandong Haineng, China). Acid detergent fiber (ADF) and neutral detergent fiber (NDF) were determined referring to the Van Soest method. Gross energy (GE), fecal energy (FE), and urinary energy (UE) were measured by an oxygen bomb calorimeter (Parr6400, Parr lnstrument Company, Champaign, IL, USA) [13]. Apparent nutrient digestibility, digestive energy (DE), methane energy (CH_4_-E), and metabolizable energy (ME) were calculated using the following formulas [14,15,16,17]:

$$\begin{array}{l} Apparent\;digestibility\;of\;nutrients\left(\%\right)\\ \;=\left(Nutrient\;content\;in\;feed-Nutrient\;content\;in\;feces\right)÷Nutrient\;content\;in\;feed\times 100\% \end{array}$$

Digestive energy (DE,MJ/d)=Intake gross energy (GE)−Fecal energy (FE)

Methane energy (CH4−E)=3.67+0.062×Intake gross energy (GE)

$$\begin{array}{l} Metabolizable\;energy\;(ME,MJ/d)\\ \;=Digestive\;energy\;(DE)-Urinary\;energy\;(UE)-Methane\;energy\;({CH}_{4}-E) \end{array}$$

### 2.6. Biochemical Indexes Analysis in Serum

The serum biochemical parameters, including glucose (GLU), triglycerides (TG), total cholesterol (TC), total protein (TP), urea, globulin (GLB), and albumin (ALB), were analyzed using an automatic biochemical analyzer (iMagic-M7, Shenzhen Courbayre, Shenzhen, China).

### 2.7. Rumen Bacterial Diversity Analysis

Total genomic DNA was extracted from the samples using a TGuide S96 Magnetic Soil/Stool DNA Kit (Tiangen Biotech (Beijing, China) Co., Ltd.) according to the manufacturer’s instructions. The quality and quantity of the extracted DNA were examined using electrophoresis on 1.8% agarose gel, and the DNA concentration and purity were determined with a NanoDrop 2000 UV–Vis spectrophotometer (Thermo Scientific, Wilmington, NC, USA). The full-length 16S rRNA gene was amplified with primer pairs 27F: AGRGTTTGATYNTGGCTCAG and 1492R: TASGGHTACCTTGTTASGACTT. It was decided to use barcoded primers, and a KOD One PCR Master Mix (TOYOBOLife Science, Osaka, Japan) was used to perform 25 cycles of PCR amplification, with initial denaturation at 95 °C for 2 min, followed by 25 cycles of denaturation at 98 °C for 10 s, annealing at 55 °C for 30 s, and extension at 72 °C for 1 min 30 s, and a final step at 72 °C for 2 min. The total PCR amplicons were purified with VAHTSTM DNA Clean Beads (Vazyme, Nanjing, China) and quantified using a Qubit dsDNA HS Assay Kit and Qubit 3.0 Fluorometer (Invitrogen, Thermo Fisher Scientific, Hillsboro, OR, USA). After the individual quantification step, amplicons were pooled in equal amounts. SMRTbell libraries were prepared from the amplified DNA by an SMRTbell Express Template Prep Kit 2.0 according to the manufacturer’s instructions (Pacific Biosciences, Menlo Park, CA, USA). Purified SMRTbell libraries from the pooled and barcoded samples were sequenced on a PacBio Sequel II platform (Beijing Biomarker Technologies Co., Ltd., Beijing, China) using a Sequel II binding kit 2.0.

### 2.8. Statistical Analysis

All data were processed by the Excel 2019 software and SPSS 21.0. All data are expressed as the means ± SEM. Differences between date means were determined using one-way ANOVA and Tukey’s multiple comparisons test. *p* < 0.05 was considered to be statistically significant. Rumen microorganism analysis was performed using BMKCloud (www.biocloud.net). The qualified sequences with more than 97% similarity thresholds were allocated to one ASVs using USEARCH (version 10.0). Taxonomy annotation of the ASVs was performed based on the Naive Bayes classifier in QIIME2 [18] using the SILVA database [19] (release 138.1) with a confidence threshold of 70%. Alpha was performed to identify the complexity of species diversity of each sample utilizing the QIIME2 2020.6 software [20]. Beta diversity calculations were analyzed by principal coordinate analysis (PCoA) to assess the diversity in the samples for species complexity. PCoA and the analysis of similarity in QIIME were used to estimate differences in bacterial communities between samples [21]. Raw sequencing reads were deposited into the National Center for Biotechnology Information Sequence Read Archive (SRA) database (accession number: PRJNA1189679).

## 3. Results

### 3.1. Apparent Digestibility of Nutrients

As shown in Table 2, the apparent digestibility of DM and CP were high in the HP and EHP groups compared to the other experimental groups (*p* < 0.05). However, there was no statistically significant difference in the DM and CP digestibility observed between the HP and EHP groups (*p* > 0.05). Furthermore, with the increase in the dietary protein level, the ADF apparent digestibility increased (*p* < 0.05), and the NDF apparent digestibility first increased and then plateaued (*p* < 0.05).

### 3.2. Nitrogen and Energy Metabolism

With the increase in the protein level in the diet (Table 3), there was an observed increase in the nitrogen intake (NI) of the rams, displaying a positive correlation with the protein levels (*p* < 0.05). Notably, a significant difference was noted among the four protein groups (*p* < 0.05), with the EHP group exhibiting the highest nitrogen intake at 15.22 g/d. FN, UN, and NR demonstrated notable increments with the escalation in protein levels (*p* < 0.05), increasing by 27.6%, 29.3%, and 77.5%, respectively. With the increase in protein levels, the ratio of FN/NI displayed a decreasing trend (*p* < 0.05), while that of MN/NI showed an increasing trend (*p* < 0.05).

With the increase in the protein level in the diet (Table 3), there was an observed increase in the nitrogen intake (NI) of the rams, displaying a positive correlation with the protein levels (*p* < 0.05). Notably, a significant difference was noted among the four protein groups (*p* < 0.05), with the EHP group exhibiting the highest nitrogen intake at 15.22 g/d. FN, UN, and NR demonstrated notable increments with the escalation in protein levels (*p* < 0.05), increasing by 27.6%, 29.3%, and 77.5%, respectively. With the increase in protein levels, the ratio of FN/NI displayed a decreasing trend (*p* < 0.05), while that of MN/NI showed an increasing trend (*p* < 0.05).

As shown in Table 4, there were no significant differences in gross energy (GE), fecal energy (FE), and methane energy (CH_4_-E) among the different groups (*p* > 0.05). With the increase in the protein level, the value of UE increased (*p* < 0.05). The metabolizable energy (ME) and DE were high in the HP group compared to the other experimental groups (*p* < 0.05). Furthermore, the HP group exhibited significantly higher gross energy digestibility (63.70%) and gross energy metabolic rate (52.56%) compared to the EHP group (*p* < 0.05).

### 3.3. Serum Biochemical Indicators

An analysis of serum biochemical parameters in the rams was conducted, as presented in Table 5. The findings indicated that with the increase in the protein level, TP and GLB first decreased and then increased (*p* > 0.05), while ALB and GLU first increased and then decreased (*p* > 0.05). Urea increased with the increase in the protein level (*p* < 0.05). The MP group exhibited higher TG (0.36 mmol/L) and TC (1.92 mmol/L) compared to the other groups (*p* > 0.05).

### 3.4. Rumen Microbial Diversity and Composition

#### 3.4.1. Alpha Diversity Analysis

**Figure 1:**
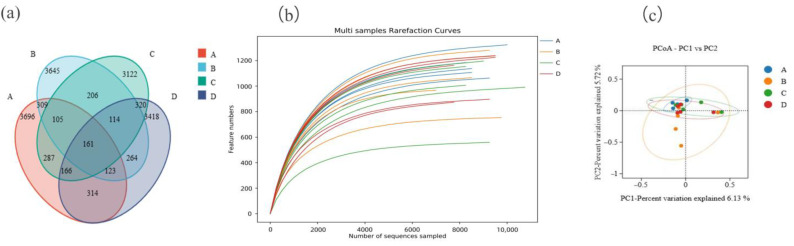
(**a**) Comparison of the differences in the Venn diagrams between the four groups. (**b**) Comparison of the differences in the dilution curves between the four groups. (**c**) Comparison of the differences in the PCoA plots between the four groups. (A, B, C, and D represent the LP group, MP group, HP group, and EHP group, respectively).

A total of 257,555 raw reads were obtained by sequencing the 16S rRNA V3~V4 region of 20 samples in the four groups in this experiment, with an average of 12,878 reads for each sample. The alpha diversity index of the samples was evaluated using the QllME2 2020.6 software. It can be seen that a total of 16,250 ASVs were obtained from the samples of the four groups at the 97% species similarity level, of which 161 were shared by the four groups, accounting for 0.99% of the total number of ASVs. There were 3696 unique ASVs in the LP group, 3645 unique ASVs in the MP group, 3122 unique ASVs in the HP group, and 3418 unique ASVs in the EHP group, accounting for 22.75%, 22.43%, 19.21%, and 21.03% of the total ASVs, respectively (Figure 1a).

The dilution curve basically tends to be gentle, indicating that the sequencing depth was reliable, which can truly reflect the composition of most microorganisms in the samples and can be used for microbial diversity analysis (Figure 1b).

**Figure 2:**
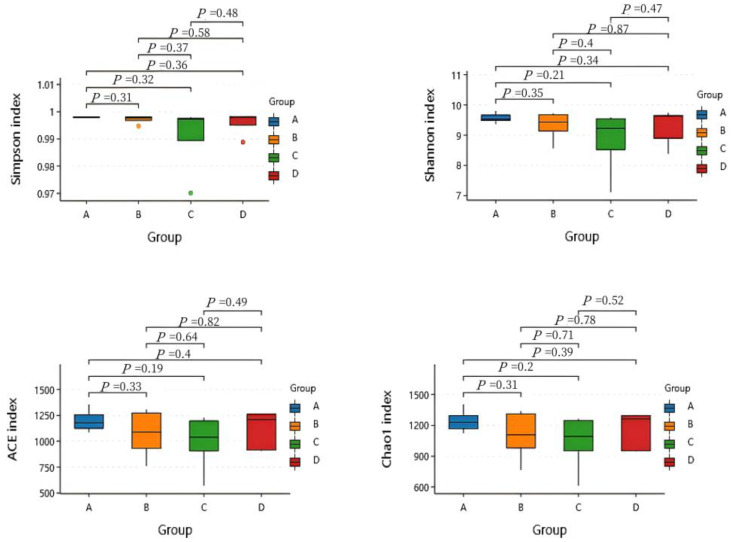
Comparison of the differences in alpha diversity between the four groups. A, B, C, and D represent the LP group, MP group, HP group, and EHP group, respectively.

It could be seen that the Shannon, Simpson, Ace, and Chao1 indices of the rumen microorganisms of the four groups were not significant (*p* > 0.05) (Figure 2).

#### 3.4.2. Beta Diversity Analysis

The PCoA cluster analysis for bacterial ASVs in the rumen fluid of the four groups was shown in Figure 1c. It can be seen that there were differences in rumen microorganisms between the MP group and the other groups.

#### 3.4.3. Effect of Different Protein Levels in Diet on Rumen Bacterial Taxonomic Composition and Community Structure (Phylum Level) in Fat-Tailed Sheep

**Figure 3:**
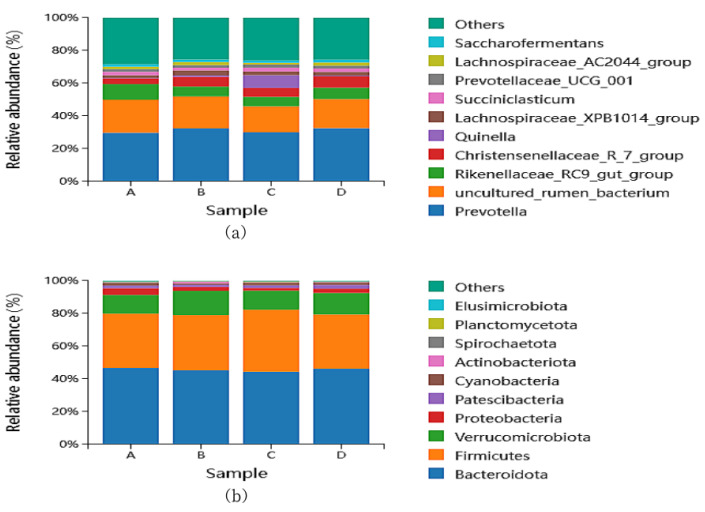
Bacterial comparisons of the rumen in Lanzhou fat-tailed sheep in the four groups (A = LP, B = MP, C = HP, D = EHP). (**a**) At the phylum level, (**b**) at the genus level.

At the phylum classification level, the top 10 species in terms of the relative abundance of rumen microbial communities in the four groups were counted (Table 6). Among these, Bacteroidota displayed the highest relative abundance in all groups, closely followed by Firmicutes. With the increase in the protein level, the relative abundance of Proteobacteria decreased first and then increased, while Verrucomicrobiota increased first, then decreased, and then increased. There was no significant difference in microbial relative abundance among the four groups (*p* > 0.05) (Figure 3a).

#### 3.4.4. Effect of Different Protein Levels in Diet on Rumen Bacterial Taxonomic Composition and Community Structure (Genus Level) in Fat-Tailed Sheep

At the genus classification level, the top 10 species in terms of the relative abundance of rumen microbial communities of the four groups were counted (Table 7). Among these, Prevotella exhibited the highest relative abundance in the four groups, at a level of 29.85%, 32.74%, 29.93%, and 32.90%. The relative abundances of uncultured_rumen_bacterium and Rikenellaceae_RC9_gut_group showed a trend of first decreasing and then increasing, while Christensenellaceae_R_7_group and Lachnospiraceae_XPB1014_group showed a trend of first increasing and then decreasing. There was no significant difference in microbial relative abundance among the four groups (*p* > 0.05) (Figure 3b).

## 4. Discussion

### 4.1. Effect of Dietary Protein Level on Apparent Digestibility of Nutrients

The level of protein in the diet directly affects the growth and production performance of animals. Studies have shown that increasing the dietary protein level can significantly improve the apparent digestibility of nutrients [22,23]. Lee et al. [24] found that the digestibility of a high-protein group was significantly higher than that of a low-protein group. In the current study, with the increase in the dietary protein level, the apparent digestibility of nutrients, such as DM, CP, ADF, and NDF, of the rams showed an increasing trend. This phenomenon primarily arises from the reduced availability of nitrogen sources for rumen microorganisms in low-protein diets. Such limitations fail to satisfy the normal growth and metabolic requirements of these microorganisms, which subsequently leads to a diminished capacity for digestion and nutrient absorption in animals [25]. In addition, the LP group had the highest corn content, and the high starch content may have reduced the pH value of the rumen, thereby reducing the apparent digestibility of nutrients [26]. Consequently, the apparent digestibility of nutrients in the low-protein diets was lower compared to that in the high-protein diets.

### 4.2. Effect of Dietary Protein Level on Energy Metabolism

After the body absorbs nutrients from the outside world, the chemical energy contained in them is released through decomposition and absorption in the body to synthesize ATP to supply energy requirements in life activities. In addition to the energy in the diet of ruminants for their own life activities, the remaining energy is lost in the form of fecal energy, urine energy, methane energy, etc. The energy derived by sheep is influenced by their diet, with the efficiency of energy utilization being closely linked to the composition of the diet as well as the digestion and utilization processes within the animal [27,28]. In this experiment, with the increase in the dietary protein level, there was no significant difference in FE and CH_4_-E between the Lanzhou fat-tailed sheep. The FE of sheep mainly changes with the change in GE intake [29], and CH_4_-E is related to the concentrate–roughage ratio of the diet [30]. Therefore, the difference between FE and CH_4_-E is not significant when the total energy difference is not significant and the ratio of dietary concentrate to concentrate is the same. This study found that as the dietary protein level increased, the UE of the rams also increased. Furthermore, the apparent digestibility and apparent metabolic rate of GE initially rose and then declined, with the highest occurring in the HP group. This pattern occurred because urinary energy increased alongside a rising nitrogen content in the urine, which was correlated with higher dietary protein levels. Additionally, the excessive absorption of protein leads to increased energy expenditure, resulting in a decrease in the digestibility and utilization rate of total energy [31,32]. In this study, the protein levels in the EHP group may have been too high, resulting in a decrease in DE/GE and ME/GE.

### 4.3. Effect of Dietary Protein Level on Nitrogen Metabolism

The dietary protein level has a great influence on nitrogen metabolism. Nitrogen intake, fecal nitrogen, urinary nitrogen, and nitrogen absorption were increased with the increase in dietary crude protein [33]. This was consistent with the results of this study, which indicated that increasing the level of dietary protein could improve the digestibility and deposition rate of nitrogen in rams and promote nitrogen deposition; however, when the dietary protein level is too high, it can significantly increase digestible nitrogen, which can lead to some nitrogen not being absorbed and utilized, which will be discharged through fecal nitrogen and urine nitrogen and finally lead to a reduction in or stabilization of nitrogen digestibility and metabolic rate [4]. Moreover, the large amount of ammonia produced by microbial-mediated deamination in rumen is one of the main reasons for the low nitrogen utilization of ruminants. Part of the NH_3_-N is used by microorganisms, part of which is used to synthesize urea in the liver through the hepatic portal vein, and most of which is excreted in the urine and wasted [31]. Therefore, reducing urinary nitrogen increases nitrogen in the digestive tract. The nitrogen content is an effective way to increase nitrogen utilization in ruminants. In this study, the urinary energy and nitrogen retention increased with the increase in the protein level. Furthermore, the HP group exhibited significantly higher gross energy digestibility (63.70%) and gross energy metabolic rate (52.56%) compared to the EHP group, implying that the HP level could effectively promote nitrogen deposition and utilization and enhance the ability of dietary energy intake of Lanzhou fat-tailed sheep. Nevertheless, the total nitrogen utilization was complex, and the specific mechanisms governing these effects warrant further in-depth investigation. While our initial findings showed that the HP protein level could improve the digestibility and deposition rate of nitrogen and promote nitrogen deposition, comprehensive testing is imperative through MCP, NH_3_-N, VFA, and other indicators.

### 4.4. Effect of Dietary Protein Level on Serum Biochemical Indexes

Biochemical analysis provides reliable information on the health condition of sheep. It is an important variable in the assessment of the adaptive and productive capacity of breeds under unfavorable environmental conditions [34]. The dietary protein level had no significant effect on the TP, ALB, and GLB of the rams. With the increase in the protein level in the diet, the urea in the serum increased significantly, which was mainly due to the fact that the protein in the diet was degraded into ammonia nitrogen through the rumen nitrogen circulation, absorbed, and transported to the liver through blood circulation to be converted into urea [35]. A high-protein diet increases the excretion of urea nitrogen because urea is the end product of protein breakdown and appears in serum and urine shortly after the ingestion of protein [36]. However, despite the increased urea nitrogen concentration, nitrogen utilization and deposition efficiency may also increase, possibly because a high-protein diet provides more essential amino acids, thus promoting nitrogen utilization and muscle growth [37].

### 4.5. Effects of Dietary Protein Level on Rumen Microbial Diversity and Community Structure

#### 4.5.1. Effects of Dietary Protein Level on Rumen Microbial Diversity of Lanzhou Fat-Tailed Sheep

Microbial alpha diversity generally refers to the number and richness of microorganisms in a particular region or ecosystem. The ASV richness was determined by the Shannon, Simpson, Chao1, abundance-based coverage estimator (ACE), and PD_whole_tree indices based on the total number of species [38]. Studies have shown that increasing the dietary protein level will reduce the diversity of rumen microorganisms [39]. According to the results of the alpha diversity analysis, the Shannon, Simpson, Ace, and Chao1 indexes of the four groups were not significant. This may mean that changing the dietary protein level has no effect on the structure and diversity of the rumen microbial community in fat-tailed sheep. The main reason is that the rumen microbial community of sheep has a certain resistance and stability, which can resist the influence caused by adjusting the dietary protein level and ensure the stability of rumen microorganisms [40].

#### 4.5.2. Effects of Dietary Protein Level on Rumen Bacterial Taxonomic Composition and Community Structure

This study revealed that the phyla Bacteroidota and Firmicutes were the predominant bacteria in the rumen of the fat-tailed sheep, which was consistent with previous studies [41,42]. This is a common feature observed for herbivorous ungulates, since these two phyla play a crucial role in fiber and carbohydrate degradation [43,44,45]. It has been proven that Firmicutes and Bacteroides are related, and an increase in one phylum level was accompanied by a decrease in the second phylum level [46] which was consistent with the results of this experiment. In this study, the level of dietary protein had no significant effect on the relative abundance of Firmicutes and Bacteroides (*p* > 0.05), but the abundance of Bacteroides in the LP group was the highest. The reason may be that Bacteroides mainly degrade fiber, and the roughage content in the LP group was the highest. An increased amount of concentrate feed in the ruminants could have contributed to higher levels of Firmicutes compared to Bacteroidetes [47]. The results of this experiment showed that the abundance of Firmicutes in the HP group was the highest, which may have been due to the high content of concentrated feed in the HP group. Proteobacteria was the phylum with the fourth-highest relative abundance in the Lanzhou fat-tailed sheep. Proteobacteria include members of many common intestinal opportunistic pathogens such as Enterobacteriaceae, Pseudomonadaceae, Moraxellaceae, etc. [48,49,50]. In this experiment, the relative abundance of Proteobacteria in the HP group was the highest, which indicates that a high-protein-level diet might inhibit the growth of harmful bacteria. At the genus level, Prevotella is one of the most important genera in rumen and is involved in carbohydrate and protein degradation [51]. Prevotella utilizes protein, starch, and hemicellulose to generate various end products [52,53]. Ellison et al. [54] found that differences in crude protein levels, neutral detergent fiber, and acidic detergent fiber in the diet caused differences in the relative abundance of Prevotella in the rumen of ruminants. In our study, we observed a higher abundance of Prevotella in the MP group than in the LP and HP groups. The results of this study may be related to the particular breed of sheep and the composition of the diet. The highest abundance of Prevotella was observed in the EHP group, indicating that the high-protein diet promoted the proliferation of Prevotella, in agreement with the study by Pang [55]. Succiniclasticum predominantly relies on starch and various other carbohydrates as its primary source of fermentation substrates. It can ferment fiber and cellulose [56]. Ma et al. [57] found that Succiniclassicum was significantly enriched in the foregut of Aohan fine-wooled sheep and was involved in the metabolism of succinate in an LEfSe analysis. At the same time, He et al. [58] observed that Succinniclassicum can utilize succinate to produce propionate, which is beneficial for intestinal health. The Succiniclasticum level was significantly increased in the HP group, indicating that a higher protein level was beneficial to the reproduction of Succiniclasticum, which corresponded to a decrease in the abundance of Proteobacteria in the HP group. Lachnospiraceae can degrade fibrous substances in the intestine to generate VFA, lower pH values, prevent harmful microbial reproduction, and maintain intestinal microbiota homeostasis. A notable finding was that the abundance of Lachnospiraceae_XPB1014_group and Lachnospiraceae_AC2044_group was higher in the rumen of the sheep in the MP group. This increase in abundance was considered to promote the development of the rumen by increasing the yield of butyrate, which directly affects the development of the rumen, improves the utilization rate of the rumen in sheep, and obtains more energy [59]. In this study, the abundance of Lachnospiraceae_XPB1014_group and Lachnospiraceae_AC2044_group in the MP group was the highest, which corresponded to the highest weight gain of the sheep in the MP group.

## 5. Conclusions

This study demonstrated that a high protein level in the diet significantly improved the apparent digestibility of nutrients and nitrogen retention and increased the urea in serum, while a low protein level improved the abundance of microorganisms. Of particular significance is the observation that a high protein (HP) level of 11.56% significantly improve the apparent digestibility of nutrients and energy use efficiency. Therefore, the optimal level of dietary protein under the conditions of this experiment was 11.56%.

## Figures and Tables

**Table 1:** The ingredients and chemical composition of the four experimental diets (dry matter basis, %).

Item	Treatments			
	LP	MP	HP	EHP
Ingredients (%)				
Wheat straw	54.00	52.00	52.70	52.00
Alfalfa hay	16.00	16.00	17.30	18.00
Corn	18.30	13.60	15.00	9.50
Soybean meal	4.60	6.00	9.70	11.50
wheat bran	5.27	8.57	3.47	7.17
Premix	1.00	1.00	1.00	1.00
Sodium bicarbonate	0.07	0.07	0.07	0.07
Salt	0.76	0.76	0.76	0.76
Nutrient level				
ME MJ/d	7.20	7.22	7.28	7.22
CP (%)	9.47	10.53	11.56	12.61
RDP (%)	5.89	6.84	7.34	8.17
RUP (%)	3.58	3.69	4.22	4.44
EE (%)	2.30	2.27	2.18	2.15
ADF (%)	39.83	40.00	39.79	40.25
NDF (%)	56.83	57.34	56.10	57.14
Calcium (%)	0.60	0.64	0.64	0.66
Phosphorus (%)	0.23	0.27	0.24	0.28

Abbreviations: LP = low protein (9.47%); MP = middle protein (10.53%); HP = high protein (11.56%); EHP = even higher protein (12.61%); premix: vitamin A, vitamin D3, vitamin E, calcium, NaCl, cobalt, iron, manganese, zinc, iodine; CP = crude protein; RDP = rumen degraded protein; RUP = rumen undegraded protein; RDP and RUP predicted from the NRC (2007) equations; ME = metabolizable energy; EE = ether extract; ADF = acid detergent fiber; NDF = neutral detergent fiber.

**Table 2:** Effect of dietary protein levels on dry matter intake, live daily gain, and apparent digestibility of nutrients in Lanzhou fat-tailed sheep.

Item	Treatment				*p*-Value
	LP	MP	HP	EHP	
Initial body weight (IBW), kg	25.0 ± 1.9	25.3 ± 2.3	25.3 ± 2.9	25.0 ± 2.3	1.000
Final body weight (FBW), kg	26.8 ± 2.2	25.9 ± 2.3	26.9 ± 2.7	26.5 ± 2.2	0.990
Live weight gain (LWG), g	31.2 ± 6.7	47.8 ± 12.3	33.1 ± 11.2	26.7 ± 8.6	0.492
Dry matter intake (DMI), kg/d	1.42 ± 0.02	1.36 ± 0.02	1.37 ± 0.02	1.40 ± 0.02	0.233
Digestibility (%)					
DM	55.75 ± 0.60 ^c^	59.96 ± 0.64 ^b^	63.89 ± 0.85 ^a^	64.40 ± 0.55 ^a^	<0.001
CP	56.71 ± 0.62 ^c^	60.52 ± 0.65 ^b^	63.92 ± 0.79 ^a^	64.02 ± 0.57 ^a^	<0.001
ADF	62.17 ± 0.48 ^c^	64.74 ± 0.65 ^b^	67.99 ± 0.96 ^a^	68.73 ± 0.77 ^a^	<0.001
NDF	65.54 ± 0.47 ^b^	66.32 ± 0.54 ^b^	68.64 ± 0.78 ^a^	68.29 ± 0.49 ^a^	<0.001

Abbreviations: LP = low protein (9.47%); MP = middle protein (10.53%); HP = high protein (11.56%); EHP = even higher protein (12.61%). Within a row values with different lowercase superscripts differ (*p* < 0.05).

**Table 3:** Effect of dietary protein levels on nitrogen utilization efficiency of Lanzhou fat-tailed sheep.

Item	Treatment				*p*-Value
	LP	MP	HP	EHP	
Nitrogen intake (NI), g/d	10.04 ± 0.05 ^d^	11.80 ± 0.04 ^c^	13.15 ± 0.02 ^b^	15.22 ± 0.04 ^a^	<0.001
Fecal nitrogen output (FN), g/d	4.34 ± 0.06 ^c^	4.66 ± 0.08 ^b^	4.78 ± 0.11 ^b^	5.50 ± 0.09 ^a^	<0.001
Urinary nitrogen output (UN), g/d	0.82 ± 0.03 ^c^	0.91 ± 0.02 ^b^	1.00 ± 0.03 ^a^	1.06 ± 0.02 ^a^	<0.001
Nitrogen retention (NR), g/d	4.88 ± 0.08 ^d^	6.23 ± 0.08 ^c^	7.37 ± 0.11 ^b^	8.66 ± 0.10 ^a^	<0.001
Efficiency					
FN/NI	0.43 ± 0.01 ^a^	0.39 ± 0.01 ^b^	0.36 ± 0.01 ^c^	0.36 ± 0.01 ^c^	<0.001
UN/NI	0.08 ± 0.00 ^a^	0.08 ± 0.00 ^a^	0.08 ± 0.00 ^a^	0.07 ± 0.00 ^b^	0.001
MN/NI	0.51 ± 0.01 ^a^	0.47 ± 0.01 ^b^	0.44 ± 0.01 ^c^	0.43 ± 0.01 ^c^	<0.001

Abbreviations: LP = low protein (9.47%); MP = middle protein (10.53%); HP = high protein (11.56%); EHP = even higher protein (12.61%); NI = nitrogen intake; FN = fecal nitrogen output; UN = urinary nitrogen output; NR = nitrogen retention; MN (metabolizable nitrogen) = FN + UN. Within a row values with different lowercase superscripts differ (*p* < 0.05).

**Table 4:** Effect of dietary protein levels on energy utilization efficiency of Lanzhou fat-tailed sheep.

Item	Treatment				*p*-Value
	LP	MP	HP	EHP	
GE intake, MJ/d	15.93 ± 0.08	15.99 ± 0.05	15.97 ± 0.02	15.84 ± 0.04	0.144
FE output, MJ/d	6.01 ± 0.08	5.92 ± 0.10	5.80 ± 0.14	6.08 ± 0.10	0.250
UE output, MJ/d	0.32 ± 0.00 ^c^	0.36 ± 0.00 ^b^	0.41 ± 0.00 ^a^	0.41 ± 0.00 ^a^	<0.001
CH_4_-E, MJ/d	1.37 ± 0.00	1.37 ± 0.00	1.37 ± 0.00	1.37 ± 0.00	0.415
DE, MJ/d	9.92 ± 0.11 ^ab^	10.07 ± 0.10 ^ab^	10.18 ± 0.14 ^a^	9.75 ± 0.10 ^b^	<0.001
ME, MJ/d	8.23 ± 0.11 ^ab^	8.33 ± 0.10 ^a^	8.40 ± 0.14 ^a^	7.98 ± 0.10 ^b^	0.01
DE/GE, %	62.24 ± 0.52 ^ab^	62.96 ± 0.59 ^ab^	63.70 ± 0.87 ^a^	61.57 ± 0.59 ^b^	0.02
ME/GE, %	51.62 ± 0.54 ^ab^	52.11 ± 0.59 ^ab^	52.56 ± 0.87 ^a^	50.37 ± 0.59 ^b^	0.02

Abbreviations: LP = low protein (9.47%); MP = middle protein (10.53%); HP = high protein (11.56%); EHP = even higher protein (12.61%); GE = gross energy; FE = fecal energy; UE = urinary energy; CH_4_-E (methane energy) = 3.67 + 0.062 × Intake gross energy (GE); DE = digestive energy; ME = metabolizable energy. Within a row values with different lowercase superscripts differ (*p* < 0.05).

**Table 5:** Effects of diets with different protein levels on serum biochemical indicators of Lanzhou fat-tailed sheep.

Item	Treatment				*p*-Value
	LP	MP	HP	EHP	
Total protein, g/L	71.24 ± 2.31	70.42 ± 2.52	66.36 ± 1.82	70.90 ± 1.05	0.314
Albumin, g/L	25.04 ± 0.47	25.28 ± 0.61	23.56 ± 0.65	25.90 ± 1.12	0.202
Globulin, g/L	46.20 ± 2.25	45.74 ± 1.83	42.20 ± 1.87	45.00 ± 1.57	0.466
White sphere ratio	0.55 ± 0.03	0.55 ± 0.02	0.56 ± 0.04	0.58 ± 0.04	0.908
Urea, mmol/L	3.30 ± 0.44 ^d^	4.62 ± 0.21 ^c^	6.85 ± 0.22 ^b^	8.06 ± 0.47 ^a^	<0.001
Glucose, mmol/L	2.07 ± 0.21	2.23 ± 0.18	2.05 ± 0.08	1.99 ± 0.22	0.805
Total cholesterol, mmol/L	1.74 ± 0.11	1.92 ± 0.29	1.56 ± 0.16	1.83 ± 0.25	0.670
Triglycerides, mmol/L	0.35 ± 0.01	0.36 ± 0.01	0.34 ± 0.01	0.32 ± 0.02	0.280

Abbreviations: LP = low protein (9.47%); MP = middle protein (10.53%); HP = high protein (11.56%); EHP = even higher protein (12.61%). Within a row values with different lowercase superscripts differ (*p* < 0.05).

**Table 6:** Relative abundance of rumen microorganisms at phylum level (%).

Phylum	LP	MP	HP	EHP	*p*-Value
Bacteroidota	46.51 ± 2.25	45.39 ± 1.88	44.62 ± 3.28	46.45 ± 2.84	0.947
Firmicutes	33.47 ± 2.38	33.85 ± 1.32	37.59 ± 4.91	32.68 ± 4.47	0.776
Verrucomicrobiota	11.43 ± 0.49	14.84 ± 1.58	11.73 ± 1.32	13.30 ± 1.46	0.253
Proteobacteria	4.16 ± 0.96	2.34 ± 0.73	1.71 ± 0.50	2.62 ± 0.66	0.152
Patescibacteria	1.57 ± 0.23	1.30 ± 0.27	1.67 ± 0.23	2.44 ± 0.89	0.434
Cyanobacteria	1.65 ± 0.34	0.78 ± 0.19	1.57 ± 0.52	1.42 ± 0.31	0.337
Actinobacteriota	0.09 ± 0.05	0.92 ± 0.81	0.08 ± 0.05	0.23 ± 0.11	0.435
Spirochaetota	0.46 ± 0.22	0.15 ± 0.05	0.25 ± 0.06	0.31 ± 0.10	0.408
Planctomycetota	0.18 ± 0.09	0.21 ± 0.06	0.27 ± 0.10	0.17 ± 0.08	0.855
Elusimicrobiota	0.14 ± 0.05	0.05 ± 0.03	0.10 ± 0.06	0.12 ± 0.06	0.662

Abbreviations: LP = low protein (9.47%); MP = middle protein (10.53%); HP = high protein (11.56%); EHP = even higher protein (12.61%).

**Table 7:** Relative abundance of rumen microorganisms at genus level (%).

Genus	LP	MP	HP	EHP	*p*-Value
Prevotella	29.85 ± 3.45	32.74 ± 2.26	29.93 ± 1.45	32.90 ± 3.28	0.767
uncultured_rumen_bacterium	20.20 ± 0.72	19.57 ± 2.27	16.07 ± 2.51	17.90 ± 1.29	0.415
Rikenellaceae_RC9_gut_group	9.29 ± 2.55	5.77 ± 1.20	5.80 ± 1.35	6.96 ± 1.48	0.457
Christensenellaceae_R_7_group	3.52 ± 0.89 ^b^	6.09 ± 0.56 ^ab^	5.25 ± 1.03 ^ab^	6.84 ± 1.31 ^a^	0.142
Lachnospiraceae_XPB1014_group	1.70 ± 0.57	3.24 ± 0.98	2.25 ± 0.37	2.23 ± 0.43	0.405
Quinella	0.38 ± 0.27	0.72 ± 0.30	7.61 ± 7.18	0.46 ± 0.39	0.431
Succiniclasticum	2.08 ± 0.31	1.62 ± 0.17	2.30 ± 0.63	2.01 ± 0.65	0.799
Prevotellaceae_UCG_001	1.66 ± 0.23	1.61 ± 0.21	1.84 ± 0.52	1.76 ± 0.33	0.962
Lachnospiraceae_AC2044_group	1.52 ± 0.30	1.90 ± 0.31	1.09 ± 0.27	1.82 ± 0.59	0.474
Saccharofermentans	1.41 ± 0.32	1.51 ± 0.14	1.39 ± 0.37	1.68 ± 0.28	0.896

Abbreviations: LP = low protein (9.47%); MP = middle protein (10.53%); HP = high protein (11.56%); EHP = even higher protein (12.61%). Within a row values with different lowercase superscripts differ (*p* < 0.05).

## Data Availability

Raw sequencing reads have been deposited into the National Center for Biotechnology Information Sequence Read Archive (SRA) database (accession number: PRJNA1189679).

## References

[1] Li J., Xu H., Liu X., Xu H., Cai Y., Lan X. (2020). Insight into the Possible Formation Mechanism of the Intersex Phenotype of Lanzhou Fat-Tailed Sheep Using Whole-Genome Resequencing. Animals.

[2] Wang X., Xu T., Zhang X., Geng Y., Kang S., Xu S. (2020). Effects of Dietary Protein Levels on Growth Performance, Carcass Traits, Serum Metabolites, and Meat Composition of Tibetan Sheep During the Cold Season on the Qinghai-Tibetan Plateau. Animals.

[3] Ludden P.A., Wechter T.L., Hess B.W. (2002). Effects of Oscillating Dietary Protein on Nutrient Digestibility, Nitrogen Metabolism, and Gastrointestinal Organ Mass in Sheep. J. Anim. Sci..

[4] Luo S.-F., Wang Y.-C., Wang X., Dai C.-P., Wang Q.-Y. (2022). Dietary Energy and Protein Levels on Lactation Performance and Progeny Growth of Hu Sheep. J. Appl. Anim. Res..

[5] Wang Y.C., Wang X., Li J.Z., Huang P.F., Li Y.L., Ding X.Q., Huang J., Zhu M.Z., Yin J., Dai C.P. (2023). The Impact of Lactating Hu Sheep’s Dietary Protein Levels on Lactation Performance, Progeny Growth and Rumen Development. Anim. Biotechnol..

[6] Noviadi R., Candra A.A., Putri D.D. (2022). Different Levels of Protein in Complete Feed Silage Based on Cassava Leaves on the Local Goats Performance. IOP Conf. Ser. Earth Environ. Sci..

[7] Prado I.N., Campo M.M., Muela E., Valero M.V., Catalan O., Olleta J.L., Sañudo C. (2014). Effects of Castration Age, Dietary Protein Level and Lysine/Methionine Ratio on Animal Performance, Carcass and Meat Quality of Friesian Steers Intensively Reared. Animal.

[8] Wang Y., Shelby S., Apple J., Coffey K., Pohlman F., Huang Y. (2021). Effects of Two Dietary Crude Protein Levels on Finishing Performance, Meat Quality, and Gene Expression of Market Lambs. Anim. Sci. J..

[9] da Costa N., McGillivray C., Bai Q., Chang K.-C., Wood J.D., Evans G. (2004). Restriction of Dietary Energy and Protein Induces Molecular Changes in Young Porcine Skeletal Muscles. J. Nutr..

[10] Fan Y., Wang Z., Nie H., Ma T., Wang Z., Wang F. (2020). Determination of Energy and Protein Requirements for Maintenance and Lactation of Dorper × Hu Crossbred Sheep. Small Rumin. Res..

[11] Cutrim D.O., Alves K.S., Santos R.d.C.d., da Mata V.J.V., Oliveira L.R.S., Gomes D., Mezzomo R. (2016). Body Composition and Energy and Protein Nutritional Requirements for Weight Gain in Santa Ines Crossbred Sheep. Trop. Anim. Health Prod..

[12] China’s feeding standard (NY/T816-2021). https://www.chinesestandard.net/PDF/English.aspx/NYT816-2021.

[13] Cui X., Wang Z., Yan T., Chang S., Hou F. (2023). Modulation of Feed Digestibility, Nitrogen Metabolism, Energy Utilisation and Serum Biochemical Indices by Dietary *Ligularia virgaurea* Supplementation in Tibetan Sheep. Animal.

[14] Mohammed A., Dei H.K., Wesseh A., Roessler R., Schlecht E. (2020). Processed False Yam Seed Meals in Broiler Chicken Diets: Effects on Feed Preference and Apparent Nutrient Digestibility. Trop. Anim. Health Prod..

[15] Jing X., Zhou J., Wang W., Degen A., Guo Y., Kang J., Xu W., Liu P., Yang C., Shi F. (2019). Tibetan Sheep Are Better Able to Cope with Low Energy Intake Than Small-Tailed Han Sheep Due to Lower Maintenance Energy Requirements and Higher Nutrient Digestibilities. Anim. Feed. Sci. Technol..

[16] Blaxter K.L., Clapperton J.L. (1965). Prediction of the Amount of Methane Produced by Ruminants. Br. J. Nutr..

[17] Wang C., Yan T., Xie K., Chang S., Zhang C., Hou F. (2021). Determination of Maintenance Energy Requirement and Responses of Dry Ewes to Dietary Inclusion of Lucerne Versus Concentrate Meal. Animal.

[18] Bolyen E., Rideout J.R., Dillon M.R., Bokulich N.A., Abnet C.C., Al-Ghalith G.A., Alexander H., Alm E.J., Arumugam M., Asnicar F. (2019). Author Correction: Reproducible, Interactive, Scalable and Extensible Microbiome Data Science Using Qiime 2. Nat. Biotechnol..

[19] Quast C., Pruesse E., Yilmaz P., Gerken J., Schweer T., Yarza P., Peplies J., Glöckner F.O. (2013). The Silva Ribosomal Rna Gene Database Project: Improved Data Processing and Web-Based Tools. Nucleic Acids Res..

[20] Zhou R., Wang L., Li Y., Wu H., Lu L., Zang R., Xu H. (2024). Effects of Tail Vegetable Fermented Feed on the Growth and Rumen Microbiota of Lambs. Animals.

[21] Lozupone C., Knight R. (2005). Unifrac: A New Phylogenetic Method for Comparing Microbial Communities. Appl. Environ. Microbiol..

[22] Zhu W., Xu W., Wei C., Zhang Z., Jiang C., Chen X. (2020). Effects of Decreasing Dietary Crude Protein Level on Growth Performance, Nutrient Digestion, Serum Metabolites, and Nitrogen Utilization in Growing Goat Kids (*Capra hircus*). Animals.

[23] Park J.H., Kim S.J., Kim N.Y., Jang S.Y., Lee J.W., Yun Y.S., Moon S.H. (2018). Effects of Dietary Crude Protein Levels on Intake, Digestibility, and Crude Protein Balance of Growing Korean Native Goats (*Capra hircus coreanae*). J. Anim. Plant Sci..

[24] Lee C., Hristov A.N., Heyler K.S., Cassidy T.W., Long M., Corl B.A., Karnati S.K.R. (2011). Effects of Dietary Protein Concentration and Coconut Oil Supplementation on Nitrogen Utilization and Production in Dairy Cows. J. Dairy Sci..

[25] Sniffen C., Robinson P. (1987). Protein and Fiber Digestion, Passage, and Utilization in Lactating Cows. Microbial Growth and Flow as Influenced by Dietary Manipulations. J. Dairy Sci..

[26] Zhang Z., Wang L., Li Q., Li F., Ma Z., Li F., Wang Z., Chen L., Yang X., Wang X. (2024). Effects of Dietary Forage Neutral Detergent Fiber and Rumen Degradable Starch Ratios on Chewing Activity, Ruminal Fermentation, Ruminal Microbes and Nutrient Digestibility of Hu Sheep Fed a Pelleted Total Mixed Ration. J. Anim. Sci..

[27] Yurtman I.Y., Savas T., Karaagac F., Coskuntuna L. (2002). Effects of Daily Protein Intake Levels on the Oral Stereotypic Behaviours in Energy Restricted Lambs. Appl. Anim. Behav. Sci..

[28] Pereira E.S., Lima F.W.R., Marcondes M.I., Rodrigues J.P.P., Campos A.C.N., Silva L.P., Bezerra L.R., Pereira M.W.F., Oliveira R.L. (2017). Energy and Protein Requirements of Santa Ines Lambs, a Breed of Hair Sheep. Animal.

[29] Tyrrell H., Moe P. (1975). Effect of Intake on Digestive Efficiency. J. Dairy Sci..

[30] Holter J., Young A. (1992). Methane Prediction in Dry and Lactating Holstein Cows. J. Dairy Sci..

[31] Yang Z.B., Yang W.R., Zhang C.Y., Song J.L., Jiang S.Z., Li S.Q. (2000). Tudy on the Correlation between Urinary Energy and Urinary Nitrogen Excretion of Qingshan Sheep. China Herbiv. Sci..

[32] Wang S.Y., Zhao X.Q., Liang J.C., Ouyang Y.N., Li Y.J., Xue B., Hong Q.H., Li W.J. (2021). Effect of Dietary Protein Level on Energy Metabolism of Yunnan Semi-Fine Wool Sheep with Empty Pregnancy. China Feed.

[33] Hoffman P., Esser N., Bauman L., Denzine S., Engstrom M., Chester-Jones H. (2001). Short Communication: Effect of Dietary Protein on Growth and Nitrogen Balance of Holstein Heifers. J. Dairy Sci..

[34] Bezerra L.R., Oliveira W.D., Silva T.P., Torreão J.N., Marques C.A., Araújo M.J., Oliveira R.L. (2017). Comparative Hematological Analysis of Morada Nova and Santa Inês Ewes in All Reproductive Stages. Pesqui. Veterinária Bras..

[35] Cortese M., Segato S., Andrighetto I., Ughelini N., Chinello M., Schiavon E., Marchesini G. (2019). The Effects of Decreasing Dietary Crude Protein on the Growth Performance, Feed Efficiency and Meat Quality of Finishing Charolais Bulls. Animals.

[36] Bilancio G., Cavallo P., Ciacci C., Cirillo M. (2019). Dietary Protein, Kidney Function and Mortality: Review of the Evidence from Epidemiological Studies. Nutrients.

[37] Li Z.J., Sui D., Zhou Y.X. (2014). Effect of Dietary Protein Level on Nutrient Digestion Metabolism and Serum Biochemical Indexes in Tan Sheep. Chin. J. Anim. Sci..

[38] Yuan X.-L., Cao M., Liu X.-M., Du Y.-M., Shen G.-M., Zhang Z.-F., Li J.-H., Zhang P. (2018). Composition and Genetic Diversity of the Nicotiana Tabacum Microbiome in Different Topographic Areas and Growth Periods. Int. J. Mol. Sci..

[39] Zhang X., Xu T., Wang X., Geng Y., Zhao N., Hu L., Liu H., Kang S., Xu S. (2021). Effect of Dietary Protein Levels on Dynamic Changes and Interactions of Ruminal Microbiota and Metabolites in Yaks on the Qinghai-Tibetan Plateau. Front. Microbiol..

[40] Allan K. (2009). What Is Microbial Community Ecology?. ISME J..

[41] Yu J., Li C., Li X., Liu K., Liu Z., Ni W., Zhou P., Wang L., Hu S. (2023). Isolation and Functional Analysis of Acid-Producing Bacteria from Bovine Rumen. PeerJ.

[42] Liu H., Jiang H., Hao L., Cao X., Degen A., Zhou J., Zhang C. (2021). Rumen Bacterial Community of Grazing Lactating Yaks (*Poephagus grunniens*) Supplemented with Concentrate Feed and/or Rumen-Protected Lysine and Methionine. Animals.

[43] Bird S., Prewer E., Kutz S., Leclerc L.M., Vilaça S.T., Kyle C.J. (2019). Geography, Seasonality, and Host-Associated Population Structure Influence the Fecal Microbiome of a Genetically Depauparate Arctic Mammal. Ecol. Evol..

[44] Brulc J.M., Antonopoulos D.A., Berg Miller M.E., Wilson M.K., Yannarell A.C., Dinsdale E.A., Edwards R.E., Frank E.D., Emerson J.B., Wacklin P. (2009). Gene-Centric Metagenomics of the Fiber-Adherent Bovine Rumen Microbiome Reveals Forage Specific Glycoside Hydrolases. Proc. Natl. Acad. Sci. USA.

[45] Fernando S.C., Purvis H.T., Najar F.Z., Sukharnikov L.O., Krehbiel C.R., Nagaraja T.G., Roe B.A., Desilva U. (2010). Rumen Microbial Population Dynamics During Adaptation to a High-Grain Diet. Appl. Environ. Microbiol..

[46] Jami E., White B.A., Mizrahi I. (2014). Potential role of the bovine rumen microbiome in modulating milk composition and feed efficiency. PLoS ONE.

[47] Khafipour E., Li S., Tun H.M., Derakhshani H., Moossavi S., Plaizier J.C. (2016). Effects of Grain Feeding on Microbiota in the Digestive Tract of Cattle. Anim. Front..

[48] Singh K.M., Reddy B., Patel D., Patel A.K., Parmar N., Patel A., Patel J.B., Joshi C.G. (2014). High Potential Source for Biomass Degradation Enzyme Discovery and Environmental Aspects Revealed through Metagenomics of Indian Buffalo Rumen. Biomed Res. Int..

[49] Bekele A.Z., Koike S., Kobayashi Y. (2010). Genetic Diversity and Diet Specificity of Ruminal Prevotella Revealed by 16s Rrna Gene-Based Analysis. FEMS Microbiol. Lett..

[50] Pohlin F., Frei C., Meyer L.C., Roch F.F., Quijada N.M., Conrady B., Neubauer V., Hofmeyr M., Cooper D., Stalder G. (2023). Capture and Transport of White Rhinoceroses (*Ceratotherium simum*) Cause Shifts in Their Fecal Microbiota Composition Towards Dysbiosis. Conserv. Physiol..

[51] Reigstad C.S., Kashyap P.C. (2013). Beyond Phylotyping: Understanding the Impact of Gut Microbiota on Host Biology. Neurogastroenterol. Motil..

[52] Stevenson D.M., Weimer P.J. (2007). Dominance of Prevotella and Low Abundance of Classical Ruminal Bacterial Species in the Bovine Rumen Revealed by Relative Quantification Real-Time Pcr. Appl. Microbiol. Biotechnol..

[53] Carberry C.A., Kenny D.A., Han S., McCabe M.S., Waters S.M. (2012). Effect of Phenotypic Residual Feed Intake and Dietary Forage Content on the Rumen Microbial Community of Beef Cattle. Appl. Environ. Microbiol..

[54] Ellison M., Conant G., Lamberson W., Cockrum R., Austin K., Rule D., Cammack K. (2017). Diet and Feed Efficiency Status Affect Rumen Microbial Profiles of Sheep. Small Rumin. Res..

[55] Pang K., Wang J., Chai S., Yang Y., Wang X., Liu S., Ding C., Wang S. (2024). Ruminal Microbiota and Muscle Metabolome Characteristics of Tibetan Plateau Yaks Fed Different Dietary Protein Levels. Front. Microbiol..

[56] An D., Dong X., Dong Z. (2005). Prokaryote Diversity in the Rumen of Yak (*Bos grunniens*) and Jinnan Cattle (*Bos taurus*) Estimated by 16s Rdna Homology Analyses. Anaerobe.

[57] Ma Y., Deng X., Yang X., Wang J., Li T., Hua G., Han D., Da L., Li R., Rong W. (2022). Characteristics of Bacterial Microbiota in Different Intestinal Segments of Aohan Fine-Wool Sheep. Front. Microbiol..

[58] He Q., Zou T., Chen J., He J., Jian L., Xie F., You J., Wang Z. (2021). Methyl-Donor Micronutrient for Gestating Sows: Effects on Gut Microbiota and Metabolome in Offspring Piglets. Front. Nutr..

[59] Wang K., Jiang M., Chen Y., Huang Y., Cheng Z., Datsomor O., Jama S.M., Zhu L., Li Y., Zhao G. (2024). Changes in the Rumen Development, Rumen Fermentation, and Rumen Microbiota Community in Weaned Calves During Steviol Glycosides Treatment. Front. Microbiol..

